# Hydrolysed Collagen Supplementation Enhances Patellar Tendon Adaptations to 12 Weeks’ Resistance Training in Middle‐Aged Men

**DOI:** 10.1002/ejsc.12281

**Published:** 2025-03-18

**Authors:** Christopher D. Nulty, Kieran Phelan, Robert M. Erskine

**Affiliations:** ^1^ School of Sport and Exercise Sciences Liverpool John Moores University Liverpool UK; ^2^ Department of Health and Sport Science South East Technological University Carlow Ireland; ^3^ Institute of Sport, Exercise and Health University College London London UK

**Keywords:** connective tissue, strength training, tendon stiffness, vitamin C

## Abstract

Resistance exercise (RE) with hydrolysed collagen (HC) supplementation increases collagen synthesis in young and middle‐aged populations, and further enhances tendon adaptations to chronic RE in young athletes. However, it is unknown if middle‐aged tendon can benefit from chronic RE with HC supplementation. We investigated the effects of 12‐weeks’ RE, combined with HC supplementation, on changes in patellar tendon (PT) properties in *middle‐aged men*. In a double‐blind design, 20 recreationally active men (age, 47 ± 5 years) were randomly assigned to a placebo (PLA, *n* = 11) or collagen (COL, *n* = 9) group. Both cohorts completed progressive lower‐limb RE twice weekly for 12 weeks and were supplemented post‐RE with COL (30 g HC and 50 mg vitamin C) or PLA (30.5 g maltodextrin and 50 mg vitamin C). The following were assessed before and after the 12‐week intervention: barbell back squat 10‐repetition maximum (10‐RM); vastus lateralis (VL) muscle thickness and PT cross‐sectional area (CSA at 25%, 50% and 75% tendon length) using ultrasonography; isometric knee extension maximum voluntary torque (MVT) and peak rate of torque development (pRTD), PT stiffness (*k*) and Young’s modulus (*ℰ*) using ultrasonography and isokinetic dynamometry. MVT_,_ pRTD, 10‐RM and VL thickness all increased post‐training (*p* < 0.05), but there were no group × time interactions (*p* > 0.05). Mean PT CSA increased more in COL (+6.8 ± 5.4 mm^2^) than PLA (+1.2 ± 2.1 mm^2^, group × time *p* = 0.027). Similarly, *k* and *ℰ* increased more in COL (*k*, +661 ± 331 N/mm and *ℰ,* +0.21 ± 0.13 GPa) than PLA (*k,* +247 ± 305 N/mm, group × time, *p* = 0.009 and *ℰ,* +0.09 ± 0.13 GPa, group × time, *p* = 0.018). In conclusion, 12‐weeks’ RE with 30 g HC supplementation augmented gains in PT CSA, stiffness and Young’s modulus in *middle‐aged* men.

**Summary**

- Twelve weeks’ high‐intensity resistance training enhanced muscle size as well as maximal and explosive strength in middle‐aged men, but these outcomes were unaffected by hydrolysed collagen supplementation.
- Resistance training without collagen supplementation enhanced patellar tendon stiffness and Young’s modulus but not patellar tendon cross sectional area in this under‐researched population.
- However, supplementing resistance training with 30 g hydrolysed collagen supplementation in middle‐aged men enhanced patellar tendon stiffness, Young’s modulus and cross‐sectional area more than resistance training alone.

## 1 Introduction

Tendon is a fibrous connective tissue that attaches muscle to bone. The dry mass of healthy human tendon comprises ∼60%–80% type I collagen fibres, which are arranged longitudinally in bundles (Wang 2006; Gelse et al. 2003). Functionally, tendons are responsible for force transmission from muscle to bone, which contributes to various biomechanical tasks including locomotion, and dynamic strength and power performance (Kubo et al. 2007; Maffiuletti et al. 2016). However, there is an age‐related decline in force and power production that begins in middle age (König et al. 2018; Kostka 2005; Pearson et al. 2002). Although human tendon cross‐sectional area (CSA) does not appear to be affected by age (Couppe et al. 2009; Carroll et al. 2008), middle‐aged and older tendon have been shown to have reduced collagen content (Couppe et al. 2009), as well as reduced stiffness and elastic modulus in vivo (Kubo et al. 2003; Onambele‐Pearson and Pearson 2012; Stenroth et al. 2012; Quinlan et al. 2018), which has been associated with a lower rate of torque development (RTD) in older men (Quinlan et al. 2017). These ageing‐induced adaptations may therefore have negative implications for injury susceptibility (e.g., by reducing the ability to prevent a fall) and for athletic performance (e.g., by limiting ‘explosivity’).

High‐intensity resistance exercise (RE) is an effective stimulus to remodel the morphological (size) and mechanical (stiffness) properties of human tendons (Bohm et al. 2015; Lazarczuk et al. 2022), which has the potential to attenuate declines in performance associated with middle age. These adaptations are underpinned by an increase in collagen content and cross‐linking of collagen fibrils within the tendon in response to repeated RE‐mediated collagen synthesis (Couppe et al. 2009; Kjær et al. 2009; Miller et al. 2005, 2007; Lee, Tang, et al. 2023). In older adults aged > 65 years, the patellar tendon is capable of increasing its stiffness and Young’s modulus following chronic RE (Reeves et al. 2003), although these adaptations likely occur at a slower rate compared with *young* men (Quinlan et al. 2021). However, it is not yet known how tendon adapts to chronic RE in middle‐aged men, an under‐researched population in the literature.

To further enhance tendon adaptations to chronic RE, recent studies have shown that supplementing RE with hydrolysed collagen (HC) ingestion can increase tendon CSA (Jerger et al. 2022, 2023), and tendon stiffness and Young's modulus (Lee, Bridge, et al. 2023; Lee et al. 2024). This is probably a consequence of both RE‐induced collagen synthesis (Lee, Tang, et al. 2023) and an increased bioavailability of collagen amino acids, which may stimulate collagen synthesis independently of RE (Mousavizadeh et al. 2020). However, all of these studies were performed in young participants, and older tendon may adapt differently to chronic RE with HC ingestion. We have recently shown that HC ingestion prior to RE leads to an increase in collagen synthesis in a dose–response manner in middle‐aged, resistance‐trained men (Nulty et al. 2024). In that study, 30 g HC ingestion led to a greater whole body collagen synthesis response than 15 g ingestion, which in turn was greater than the 0 g HC intervention. Interestingly, collagen synthesis did not change throughout the 0 g HC intervention, suggesting reduced sensitivity regarding a collagen synthesis response to RE in middle‐aged men. The finding that 15 g HC recovered the collagen synthesis response to RE, and that 30 g augmented this response further, highlights the importance of supplementing chronic RE with HC to potentially augment tendon adaptations in middle‐aged individuals. However, to date, no study has investigated whether HC supplementation can affect changes in tendon properties following chronic RE in this population.

Therefore, with this study, we aimed to investigate the effects of 12 weeks’ progressive lower‐limb resistance exercise (RT) combined with 30 g HC supplementation on patellar tendon stiffness, Young’s modulus and CSA in recreationally active (but naïve to resistance training) middle‐aged men. Secondly, we aimed to describe the effects of this intervention on changes in lower body strength and power, assessed by knee‐extensor maximum voluntary torque (MVT), leg press strength, countermovement jump (CMJ) height and broad jump (BJ) distance. We hypothesised that middle‐aged men supplemented with vitamin C‐enriched hydrolysed collagen would demonstrate augmented adaptations in patellar tendon properties following 12 weeks’ lower‐limb RT compared to RT alone, which would translate into improved physical performance.

## 2 Methods

### 2.1 Participants

*A priori sample size estimation:* A minimal sample size was estimated prior to conducting the study with G*Power software (version 3.1.9.6, Heinrich‐Heine‐Universität Düsseldorf, Düsseldorf, Germany). This estimation was based on the results from the only study published prior to the start of the present study that examined the effect of nutritional supplementation with RT on patellar tendon CSA (Farup et al. 2014), that is, one of the main dependent variables in the present study. These authors reported an increase in tendon CSA of 14.9% ± 3.1% and 8.1% ± 3.2% in the treatment and placebo groups, respectively (Farup et al. 2014). Thus, we used a large effect size (*f* = 0.75) to calculate the minimum sample size required for the present study, which indicated that at least 18 participants were required to detect an effect of collagen (COL) versus placebo (PLA) (two‐way analysis of variance (ANOVA); α: 0.05 and power: 0.80). To account for an expected participant withdrawal of ∼20%, and to ensure the study remained statistically powered, 24 healthy middle‐aged men, who were physically active or involved in recreational sport (> 120 min moderate activity and/or sport training per week) but naïve to lower limb resistance exercise, provided written informed consent to participate in this study. Volunteers were aged between 40 and 60 years, with no history of lower limb injury in the last 12 months. They were also required to be nonsmokers and could not be vegan or vegetarian, as the HC supplement was derived from bovine connective tissue. Participants were recruited by word of mouth as well as flyers posted at local sports clubs, on the University campus and on the University social media platforms. Four participants were unable to adhere to the training intervention due to personal commitments and were removed from the study. Therefore, 20 participants completed the study (PLA: *n* = 11, age, 46 ± 5 years, height, 181 ± 6 cm, body mass and 84 ± 12 kg; COL: *n* = 9, age, 48 ± 5 years, height, 174 ± 6 cm, body mass and 78 ± 12 kg). The study was registered at https://clinicaltrials.gov/(identifier: NCT06402890), was approved by the Ethics in Research Committee at South East Technological University (Approval No. 300/2021) and complied with the Declaration of Helsinki.

### 2.2 Experimental Design

**FIGURE 1:**
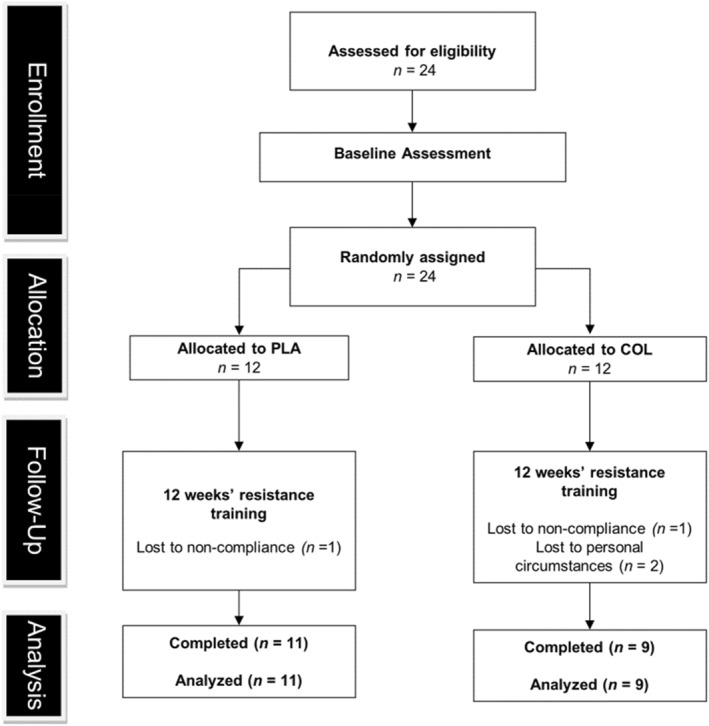
CONSORT flow diagram showing participant recruitment and intervention timeline. PLA = placebo group and COL = collagen group.

This study was a double‐blind, randomised control design with parallel groups (Figure 1). Data collection began in July 2021 and was completed in December 2021. Following familiarisation, participants were pair‐matched for age, body mass, isometric knee extension strength, barbell back squat 10 repetition maximum (10‐RM) and assigned to one of two groups (COL or PLA). Participants attended an initial visit to the laboratory where they were screened, provided with information on how to complete a 3‐day food diary, and familiarised with all the baseline measurements and procedures. As the participants were naïve to resistance exercise, each participant was provided with instruction and coaching on the back‐squat technique from an experienced sport and exercise scientist. Upon returning to the laboratory and before engaging in any physical activity, muscle architecture and patellar tendon CSA images were obtained. Participants completed a general warm‐up, comprising 5 min low‐intensity jogging and 5 min dynamic stretching before baseline measurements, which were conducted in the following order: (1) muscle architecture; (2) patellar tendon CSA; (3) bilateral vertical countermovement jump (CMJ) and broad jump; (4) tendon pre‐conditioning with 10 progressive isokinetic knee extensions/flexions at 60°·s^−1^; (5) maximal isometric knee extension and flexion assessments; (6) ramped isometric knee extension (patellar tendon mechanical properties); and (7) 10‐RM back squat. Participants then completed a 12‐week progressive lower‐limb RT programme, with two supervised RT sessions performed per week. For measurements 1 and 2, all testing was conducted on the participant's right leg. Supervised RT sessions took place twice a week and involved barbell back squats, dumbbell Romanian deadlifts, trap‐bar deadlifts and dumbbell goblet squats. Training load was adjusted and progressed weekly, based on performance in the same session the previous week. Immediately after each training session was completed, participants consumed their assigned supplement (COL or PLA) under the supervision of the researchers. Participants returned to the laboratory 72 h after completion of their final training session to repeat all baseline assessments.

### 2.3 Habitual Dietary Behaviour and Anthropometry

Height was measured using a wall‐mounted stadiometer (Model 264, Seca Gmbh&Co. Hamburg, Germany) and body mass was measured using calibrated weighing scales (Model 704 W Seca Gmbh & Co., Hamburg, Germany), with participants shoeless and wearing standard exercise clothing. During the familiarisation and baseline testing period, participants were asked to record their habitual dietary behaviour using a food and drink diary across 3 days (Thursday to Saturday). Food diaries were analysed for total energy and macro‐ and micro‐nutrient composition using the professional dietary analysis software (Nutritics Research Edition, version 5.09, Dublin, Ireland). Total daily intakes were averaged across the 3 days and normalised to body mass. Absolute and relative (to body mass) daily nutritional compositions for COL and PLA are presented in Table 1.

**TABLE 1:** Habitual energy, macronutrient and micronutrient intake assessed during the pre‐training period.

Nutritional composition	COL (*n* = 9)	PLA (*n* = 11)	*t*‐test, *p*
*Energy intake*			
Habitual intake (kcal·d^−1^)	1934 ± 648	2238 ± 494	0.303
*Carbohydrate intake*			
Habitual intake (g·d^−1^)	219 ± 112	239 ± 49	0.641
Habitual intake (g·kg·d^−1^)	2.8 ± 10.9	2.8 ± 4.8	0.675
*Protein intake*			
Habitual intake (g·d^−1^)	81.6 ± 22.7	92.6 ± 19.1	0.314
Habitual intake (g·kg·d^−1^)	1.1 ± 2.2	1.1 ± 1.9	0.328
*Fat intake*			
Habitual intake (g·d^−1^)	70.9 ± 19.4	84.3 ± 49.3	0.357
Habitual intake (g·kg·d^−1^)	0.9 ± 1.9	1.0 ± 3.2	0.606
*Vitamin C intake*			
Habitual intake (mg·d^−1^)	115 ± 37	109 ± 20	0.901
Habitual intake (mg·kg·d^−1^)	1.5 ± 3.6	1.3 ± 11.8	0.618

### 2.4 Muscle Architecture

B‐mode ultrasonography (Clear Vue 550, Koninklijke Philips, Eindhoven, The Netherlands) was used to obtain sagittal images of the *m. vastus lateralis* (VL) at 50% muscle length. This muscle was chosen due to it being representative of the *m. quadriceps femoris* (Erskine et al. 2009). Participants lay in a supine position on an elevated plinth, while all images were collected from the right leg with the knee and hip at full extension. The 38 mm wide 4–12 MHz linear transducer was placed on four sites of the skin of the right thigh to identify the origin and the distal myotendinous junction of the VL (to determine VL length) and the lateral and medial borders of the VL at 50% muscle length (to determine VL width and, ultimately, the centre of the VL muscle belly). Resting muscle architecture measurements (muscle thickness and fascicle pennation angle (*θ*_*p*_), i.e., the angle of the fascicles as they insert into the lower aponeurosis) were then obtained by taking a single image of the VL muscle belly centre. To prevent compression of the underlying muscle structures during ultrasound‐based muscle architecture measurements, the transducer was positioned without contacting the skin using a generous layer of ultrasound gel as the sole interface. Each image was manually analysed using an image analysis software by a single operator with 10 years’ ultrasound experience (Image J v1.8.0, National Institute of Health, MD, United States). Muscle thickness (MT) was computed as the mean distance between the upper and lower aponeuroses based on three perpendicular lines drawn at equal intervals along the width of the image. Fascicle pennation angle was computed from the mean angle of at least three fascicle insertions into the lower aponeurosis.

**FIGURE 2:**
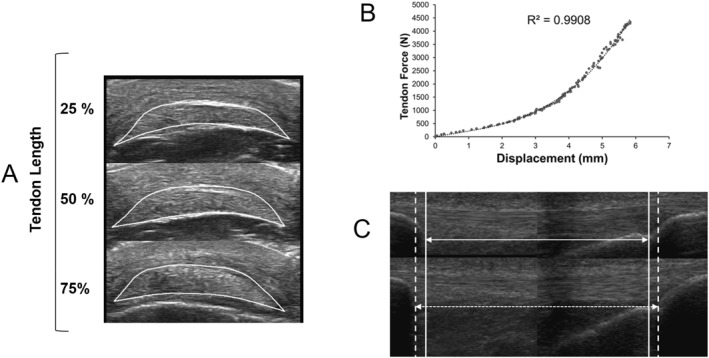
(A) Representative ultrasound image of patellar tendon (PT) cross sectional area at 25%, 50% and 75% PT length; (B) example of the force‐elongation curve during a 6 s ramped isometric maximum voluntary contraction (MVC) and (C) ultrasound image depicting changes in PT length from rest to MVC (solid arrow represents resting tendon length and dashed arrow represents tendon length at MVC). For this image, two separate ultrasound video frames from the proximal and distal ends of the tendon (taken during two separate MVCs) were joined together at a common echo‐absorbent reference point at 50% PT length. *N.B.* This image is for illustration purposes only. The method used to determine PT elongation is detailed in Section 2.6.3.

### 2.5 Patellar Tendon Morphology

Participants were securely strapped to an isokinetic dynamometer (Biodex System 3, IPRS, Suffolk, UK) in a seated position with their knee joint set at 90° knee flexion and their hip joint set at 85°. Patellar tendon (PT) morphology was assessed via B‐mode ultrasound (Clear Vue 550, Koninklijke Philips, Eindhoven, The Netherlands) with the participant at rest. PT length was defined as the distance between the patella apex and insertion point of the PT into the tibial tuberosity, where the 38 mm wide linear transducer was placed over the knee in the sagittal plane. PT cross‐sectional area was assessed at 3 points, 25%, 50% and 75% tendon length, after these locations were identified with ultrasound and marked on the skin with an indelible pen. An axial scan to produce a transverse CSA image was taken at each point ensuring all borders of the PT were clearly visible, which typically took 1–3 efforts per image (Figure 2). Each image was manually analysed by tracing the outline of PT using an image analysis software (Image J v1.8.0, National Institute of Health, MD, United States).

### 2.6 Patellar Tendon Mechanical Properties

#### 2.6.1 Maximal Isometric Strength

Maximal isometric knee extension (KE) and knee flexion (KF) torque were assessed in the seated position as described above. Participants were familiarised by performing 3 submaximal isometric KE and KF contractions. Participants were then asked to perform between 2 and 4 KE and KF maximum voluntary contractions (MVCs) and asked to ‘push’ or ‘pull’ as hard as possible while receiving constant verbal encouragement. The number of attempts was increased if the highest attempt was ≥ 10% higher than the second highest attempt. Each MVC lasted ∼2–3 s with 60 s rest between contractions. The highest KE MVC and KF MVC were used for analysis. The analogue torque signal was sampled at 2000 Hz and recorded using the commercially available data acquisition software (EMGworks Acquisition v. 4.8.0, Delsys Inc., Manchester, UK). During offline analysis, the torque was low pass‐filtered at 10 Hz, using a second‐order Butterworth filter, and corrected for gravity by subtracting the baseline torque. The torque was divided by the PT moment arm of healthy men at 90° knee flexion (Erskine et al. 2009) in order to calculate force (N).

KE rate of torque development (RTD) was also calculated from the torque‐time curve produced during MVC (where no countermovement could be detected). The time of torque onset was identified manually as the last trough in the baseline torque before an exponential increase. Torque at 50 ms, 100 ms and 150 ms after torque onset was measured, and the RTD was calculated during 0–50, 50–100 and 100–150 ms time windows by dividing the difference in torque by the difference in time. Peak RTD (pRTD) was also calculated as the steepest slope in the torque‐time curve occurring in the first 150 ms after torque onset. All RTD measures were then normalised by expressing them as a percentage of KE maximal isometric voluntary torque.

#### 2.6.2 Antagonist Co‐Activation

Hamstring activation was assessed via surface electromyography (sEMG) of the *m.* *biceps* *femoris* long head (BF) and was recorded during all contractions using a wireless EMG system (Trigno Wireless, Delsys Inc., Manchester, UK). The BF was identified by palpation during submaximal knee flexion. The skin was prepared by shaving, abrading and cleaning with alcohol. A single wireless sensor (Trigno Avanti, Delsys Inc., Manchester, UK) was attached at 2/3 muscle length from the proximal end (Freriks et al. 1999). To ensure synchronisation between measures, EMG signals were sampled using the same analogue to digital converter and the same frequency as the torque signals. During analysis, EMG signals were band‐pass filtered, using a second‐order Butterworth filter at 10–500 Hz. The root mean square (RMS) of the filtered EMG signal was measured over a 500 ms epoch around maximal torque production during KF MVC. Additionally, the torque and EMG amplitude were resampled to match the ultrasound video frequency (28–38 Hz). During the ramped contraction, antagonist torque was estimated by expressing the KF BF amplitude relative to the BF amplitude during KF MVC and multiplying by the maximal KF torque.

#### 2.6.3 Force–Elongation Relationship and Patellar Tendon Stiffness

In order to precondition the PT, 10 repetitions of isokinetic knee extension and flexion through the participants full range of motion were performed at 60°·s^−1^. The participant was instructed to perform the first repetition at approximately 10% of their perceived maximum effort and increase effort by 10% on every repetition before a final 10th repetition performed at maximal (100%) effort. Ramped isometric KE MVCs (RMVCs) were used to measure the PT force–elongation relationship and calculate its mechanical properties during KE. The 38 mm wide linear array transducer (Model L12‐4 [4–12 MHz] Phillips ClearVue 550, Amsterdam, NL) was not wide enough to capture the entire length of the PT in one scan. Therefore, a 2 mm wide strip of surgical tape (3M Transpore White Medical Tape, 3M, Dublin, Ireland) was placed transversely over the skin to act as an echo‐absorbent reference point at 50% tendon length. The probe was placed in the sagittal plane over the PT and held firmly in place but with minimal pressure (to avoid compressing the tendon) during each contraction. Participants performed between 2 and 5 submaximal ramped contractions until the investigator was satisfied that they could perform a smooth contraction with a constant loading rate. Participants then performed 2–3 RMVCs lasting 6 s. This process was repeated with the probe placed on the distal aspect of the PT, with both the tape and tibial tuberosity clearly visible in the video. PT elongation was measured offline, using an open‐source semi‐automatic tracking software (Tracker for Windows v. 6.0.8) to track displacement of both the PT apex and the PT insertion on the tibial tuberosity. In cases where lower‐quality frames caused the semi‐automatic tracker to lose the pixel, these frames (< 10% of ∼150 per video) were manually excluded following qualitative inspection. Concurrently, KE torque antagonist sEMG were recorded as stated above, with an additional EMG sensor attached to the ultrasound probe to provide a simultaneous signal interruption on both the ultrasound video and torque trace, allowing time synchronisation between measurements during offline analysis.

To synchronise the force and elongation measurements during the proximal and distal ultrasound videos and contractions, force‐time curves were resampled in the first instance to match the sampling frequency of each ultrasound video. Separate force–elongation curves were then constructed for the proximal and distal PT. The force readings from the weakest of the two RMVC measurements were used for analysis. Total PT elongation was calculated by summing the elongation at one end during the weakest RMVC and the interpolated elongation from the opposing end at matching force levels. For example, if proximal elongation at 1600 N was 1.5 mm, the corresponding distal elongation at 1600 N (e.g., 0.5 mm) was interpolated from the distal force–elongation curve and summed to calculate the total elongation (2.0 mm). Force was calculated from torque, as described during the maximal isometric contraction section above, and each force–elongation relationship was fitted with a second or third order polynomial (Figure 2). For each participant, patellar tendon stiffness (*K*) was calculated from the force–elongation slope, using the highest 20% absolute force values of the weakest RMVC (typically obtained pre‐training). Young’s modulus (*ℰ*) was calculated by obtaining the ratio of PT length to mean PT CSA and multiplying by the PT stiffness. PT stress was calculated by dividing maximal PT force by mean PT CSA. PT strain was expressed as a percentage of maximal PT elongation relative to PT resting length.

### 2.7 Bilateral Vertical Countermovement Jump (CMJ) and Broad Jump (BJ)

Participants performed an additional dynamic warm‐up consisting of 5 min submaximal jogging activity, 5 min dynamic stretching and 3 submaximal CMJs and BJs. For CMJ, participants were required to remove shoes and stand upright with hands remaining on hips for the entirety of the jump. They were then instructed to quickly squat to a self‐selected depth before immediately and rapidly reversing the movement into a maximal effort jump with knees fully extended. To ensure consistent jump height measurements using photoelectric cells (Optojump, Mircrogate, Bolanzo, Italy) and that participants jumped and landed in the same spot, tape marked with an ‘X’ was placed on the floor as a visual reference. During each visit, participants performed 3–5 practice submaximal jumps, receiving live feedback to reinforce proper technique. Instructions emphasised landing with knees extended and minimising excessive flexion upon landing. Valid and invalid trials were demonstrated during the familiarisation session and prior to each assessment. Three maximal CMJs were performed, separated by 1 min rest, with jump height assessed. BJs required the same start position as CMJ, and participants were asked to jump forward as far as possible, keeping the hands on the hips and land with both feet. BJ distance was measured by the placing a wooden dowel against the heels to mark the landing spot and aligned with a measuring tape on the floor. Three maximum BJ efforts were performed and separated by 1 min rest. The highest CMJ and longest BJ were used for analysis.

### 2.8 Barbell Back Squat 10‐Repetition Maximum (10‐RM)

Participants rested for 2 min after completing all jump assessments. In order to standardise depth during back squats, participants were asked to place the 20 kg Olympic barbell on their shoulders and squat down until they reached a depth equivalent to 90° knee flexion, which was determined by the researcher using a goniometer. A box was placed at this height to allow participants to touch briefly on each repetition. One set of the barbell back squats with no additional load was performed initially, followed by 1 set of 10 repetitions at 50% of their estimated 10‐RM. The load was then increased by 5–20 kg on each subsequent set for a total of 4–6 attempts, separated by 3 min rest until the participant could only perform a maximum of 10 repetitions. 10‐RM was deemed to be the load used during the final set where the participant successfully completed 10 repetitions.

### 2.9 Resistance Training

All participants completed two resistance training sessions per week (separated by 48–72 h) for 12 weeks, resulting in a total of 24 sessions (100% participant compliance), with the aim of overloading the lower limbs, particularly the quadriceps MTU. Participants began the first training session working at a load of 90% 10‐RM. The objective during each session was to complete at least 8, but no more than 10 repetitions for all 4 sets, each set separated by 3 min rest, with participants instructed to terminate sets at 10 repetitions or concentric failure depending on which came first. Participants were instructed to perform the eccentric portion under control over ∼2 s duration, and the range of motion was standardised for each exercise, with participants instructed to lightly touch a tactile indicator (e.g., weight bench and plates for squat depth). Each exercise was progressed independently, and load was determined by performance in the same session the previous week, that is, the external load was increased by 2.5–5 kg when the participant could successfully complete 4 sets of 10 repetitions. This procedure applied to all exercises for the first 6 weeks’ training. For the second 6 weeks, all exercises and rest periods remained the same; however, the objective for each session was to complete at least 6 repetitions but no more than 8 repetitions during all 4 sets. This was done to ensure continuous overload and limit the chance of plateauing in volume and intensity. Participants then progressed each exercise by adding 2.5–5 kg each week after successful completion of 4 sets of 8 repetitions during the corresponding session during the previous week.

### 2.10 Nutritional Supplementation

The dry ingredients for both the hydrolysed collagen supplement (COL) and maltodextrin placebo (PLA) were prepared into sachets by a laboratory technician independent of the study. The sachets were stored in containers identifiable by red and green coloured labels and matched pairs of participants were placed into ‘Red’ and ‘Green’ groups to ensure double blinding of the participants and investigators. The composition of each supplement is included in Table S1. The COL contained 30 g unflavoured hydrolysed collagen (Collagen Protein, MyProtein, Manchester, UK), 50 mg vitamin C powder (Holland and Barrett, Dublin, Ireland) and 3 g noncaloric sweetener (Pure Via 100% Xylitol, Mersiant UK LTD., Buckinghamshire, UK). The PLA was matched for caloric value, vitamin C content, taste and colour and contained 30.5 g of maltodextrin (MyProtein, Manchester, UK), 50 mg vitamin C powder and 4 g of noncaloric sweetener. As soon as each training session ended, participants were instructed to collect the coloured sachet of dry supplement ingredients from the sample box corresponding to their colour group allocation. Each participant was provided with their own opaque bottle and mixed the dry ingredients with 400 mL water, which they consumed under supervision within 5 min of completing their training session. Supplement intake only took place on training days and was supervised to ensure compliance. Outside of the supervised training sessions, participants were instructed to maintain their habitual dietary habits for the duration of the study.

### 2.11 Statistical Analysis

Data are reported as mean ± standard deviation unless stated otherwise. All data were analysed using the statistical software package for social sciences (SPSS v. 28, IBM, Armonk NY, United States), with significance accepted at *p* < 0.05. Independent *t*‐tests were performed on all variables between COL and PLA groups to assess whether there were any differences at baseline. Adaptations to the training and nutrition intervention were assessed for main effects and training × group interaction effects by two‐way analysis of variance (ANOVA). When significant main effects or interaction effects were observed, paired *t*‐tests were used for post hoc pairwise comparisons. Independent *t*‐tests were used to compare the absolute pre‐to‐post differences between groups. Changes in PT CSA were assessed for main effects of training and location (i.e., the location along its length relative to tendon origin), as well as any interactions between training, group, and location by three‐way ANOVA, with paired *t*‐tests used for post hoc pairwise comparisons. Partial eta squared (*η*_*p*_^2^) for ANOVA effects and Cohen’s d for *t*‐tests were reported as effect size estimates for each corresponding statistical model. The thresholds for *η*_*p*_^2^ and Cohen’s d are categorised as small (*η*_*p*_^2^ = 0.01 and *d* = 0.20), medium (*η*_*p*_^2^ = 0.06 and *d* = 0.50) and large (*η*_*p*_^2^ = 0.14 and *d* = 0.80) (Cohen 2013).

## 3 Results

### 3.1 Dietary Intake and Body Mass

There were no differences in absolute intake nor intake relative to body mass of macronutrients or vitamin C between COL and PLA during the pre‐intervention period (Table 1, *p* > 0.05). There were no differences in body mass between groups pre‐intervention (*t*_18_ = 1.16 and *p* = 0.261) and there were no changes following the intervention (F_1,18_ = 2.87 and *p* = 0.109).

### 3.2 Muscle Strength, Power and Architecture

At baseline, there were no differences in KE MVT (*t*_18_ = 0.323 and *p* = 0.751) or pRTD (*t*_18_ = −0.197 and *p* = 0.846) between groups. Changes in isometric strength, VL muscle architecture and jump performance following 12 weeks’ RT are presented in Table 2. All variables except for VL fascicle pennation angle improved following the intervention (*p* < 0.05), but there were no group × time interactions for any of the measures (*p* > 0.05).

**TABLE 2:** Muscle function and architecture adaptations to 12 weeks’ resistance training. Data are mean ± SD.

	PLA		COL		
Variable	Pre	Post	Pre	Post	G × T, *P*
KE MVT (N·m)	232 ± 56	247 ± 46*	213 ± 62	233 ± 44*	0.754
10‐RM (kg)	67 ± 16	97 ± 18*	72 ± 14	109 ± 19*	0.116
KE pRTD (N·m^.^s^−1^)	990 ± 479	1215 ± 404*	1038 ± 582	1211.7 ± 577*	0.637
KE pRTD (% MVT)	411 ± 160	500 ± 155*	468 ± 238	532 ± 240*	0.581
BJ distance (cm)	140 ± 30	143 ± 32*	149 ± 29	156 ± 26*	0.552
CMJ height (cm)	25.1 ± 5.2	26.9 ± 6.4*	26.3 ± 5.6	28.7 ± 6.8*	0.589
CMJ power (W)	3272 ± 626	3382 ± 689*	3065 ± 499	3221 ± 563*	0.422
VL MT (mm)	24.8 ± 3.2	25.1 ± 2.4*	23.4 ± 3.2	24.5 ± 2.9*	0.714
VL *θ*_*p*_ (°)	16.2 ± 3.1	16.8 ± 2.9	16.6 ± 2.8	17.2 ± 1.5	0.934

Abbreviations: *θ*_*p*_, fascicle pennation angle; 10‐RM, leg press 10 repetition maximum; BJ, broad jump; CMJ, countermovement jump; KE, knee extension; MT, muscle thickness; MVT, maximum voluntary torque; pRTD, peak rate of torque development; and VL, vastus lateralis.

*Greater than pre‐training (p < 0.05).

### 3.3 Explosive Torque Production and Rate of Torque Development (RTD)

Absolute and normalised explosive torque production at 50, 100 and 150 ms after the onset of torque were not different between COL and PLA at baseline (*p* > 0.05). Similarly, there were no between group differences in absolute or normalised pRTD at baseline (*t*_17_ = −0.197, *p* = 0 0.846 and *t*_17_ = −0.197, *p* = 0.846), which occurred at 78 ± 20 ms after torque onset. Increases in pRTD post‐intervention are shown in Table 2, but there was no group × time interaction (F_1,17_ = 0.231 and *p* = 0.637). Both absolute (time, F_1,17_ = 6.366, *p* = 0.022 and *η*_*p*_^2^ = 0.272) and relative (time, F_1,17_ = 6.6719, *p* = 0.020 and *η*_*p*_^2^ = 0.294) RTD increased following the intervention, but there was no group × time interaction (F_1,17_ = 0.141 and *p* = 0.712). There was also an effect of time window, where RTD was highest 50–100 ms after torque onset (F_1,17_ = 34.077, *p* < 0.001 and *η*_*p*_^2^ = 0.667).

**TABLE 3:** Patellar tendon mechanical properties before (Pre) and after (Post) 12‐week’ RT with (COL) and without (PLA) hydrolysed collagen supplementation.

	PLA		COL		
Variable	Pre	Post	Pre	Post	G × T, *P*
Stiffness (N/mm)	1304 ± 622	1551 ± 612*	1174 ± 294	1836 ± 518*	0.009
Young's modulus (GPa)	0.53 ± 0.29	0.62 ± 0.29*	0.52 ± 0.14	0.76 ± 0.17*	0.018
Stress (MPa)	25.5 ± 8.5	42.1 ± 9.6*	34.2 ± 9.6	43.3 ± 7.3*	0.103
Elongation (mm)	4.8 ± 1.7	4.6 ± 1.2	5.7 ± 1.8	4.6 ± 1.1*	0.053
Strain (%)	10.7 ± 4.0	9.9 ± 2.6	11.3 ± 3.4	9.1 ± 2.3*	0.082
Mean tendon CSA (mm^2^)	117 ± 6	117 ± 6	108 ± 13	115 ± 10*	0.027
RMVC peak tendon force (N)	2956 ± 990	3658 ± 1041*	3408 ± 1026	3963 ± 1157*	0.288

*Note:* Data are mean ± SD. PLA, placebo group and COL, hydrolysed collagen group. *Different to pre‐training (*p* < 0.05).

**FIGURE 3:**
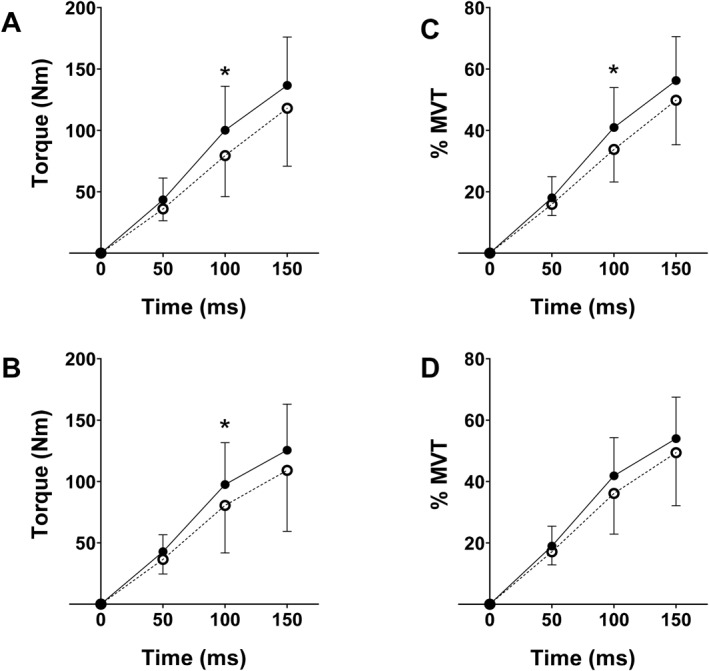
Absolute explosive torque before (dashed line) and after (solid line) 12 weeks’ resistance training without (A, PLA, *n* = 10) and with 30 g hydrolysed collagen supplementation (B, COL, *n* = 9) and explosive torque normalised to maximum voluntary torque (MVT) before (dashed line) and after (solid line) 12 weeks’ resistance training without (C, PLA, *n* = 10) and with 30 g hydrolysed collagen supplementation (D, COL, *n* = 9).

Absolute explosive torque increased at 100 ms (F_1,17_ = 15.022, *p* = 0.001, *η*_*p*_^2^ = 0.484) and 150 ms (F_1,17_ = 8.543, *p* = 0.010,* η*_*p*_^2^ = 0.348) after the onset of torque following the 12‐week’ RT, respectively, but not at 50 ms after torque onset (F_1,17_ = 4.259, *p* = 0.056). Normalised explosive torque increased post‐training at 100 ms (F_1,17_ = 5.672, *p* = 0.030, *η*_*p*_^2^ = 0.262) but not at 50 (F_1,17_ = 1.226 and *p* = 0.285) nor 150 ms (F_1,17_ = 2.894, *p* = 0.108) after torque onset (Figure 3).

**FIGURE 4:**
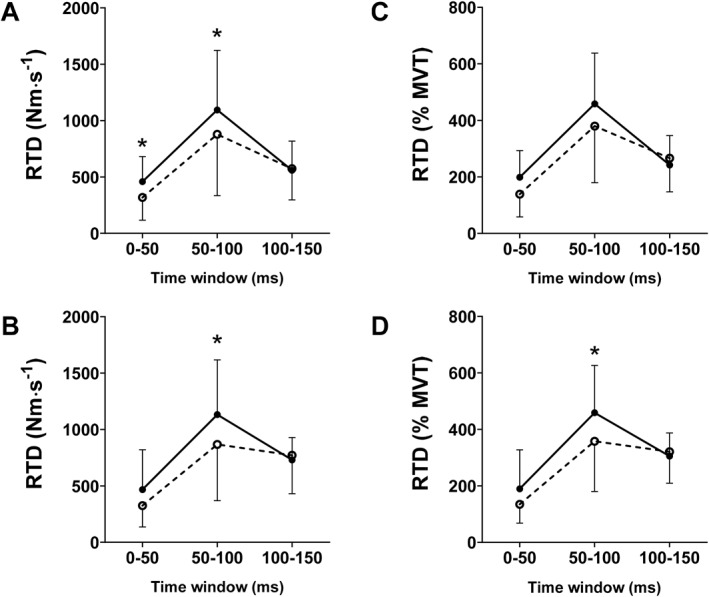
Absolute rate of torque development (RTD) before (dashed line) and after (solid line) 12 weeks’ resistance training with (A, COL = 9) without 30 g hydrolysed collagen supplementation (B, PLA, *n* = 10) and normalised RTD before (dashed line) and after (solid line) 12 weeks’ resistance training with (C, COL, *n* = 9) and without 30 g hydrolysed collagen supplementation (D, PLA, *n* = 10).

However, regarding absolute RTD and RTD normalised to MVT, there were no interactions between time × time window or time × time window × group (*p* > 0.05), and for absolute and normalised explosive torque and RTD, there were no interactions between time × group (*p* > 0.05). Pre‐to post‐training changes in explosive torque and RTD are displayed in Figure 4.

### 3.4 Patellar Tendon Cross Sectional Area

**FIGURE 5:**
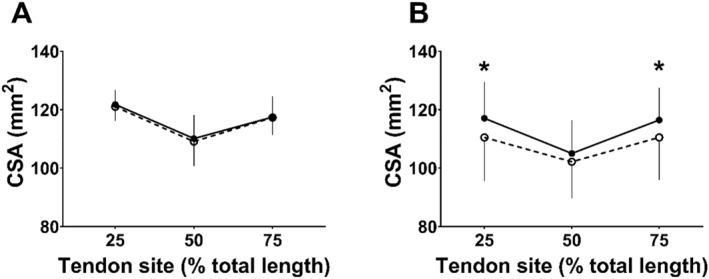
Patellar tendon cross sectional area (mm^2^) before (open circles) and after (black circles) 12 weeks’ resistance training in A. PLA (*n* = 11) and B. COL (*n* = 9). *Greater than pre (*p* < 0.05).

At baseline, there were no between group differences in mean PT CSA (*p* = 0.064). However, CSA was not the same at each location along the tendon length [main location effect (F_2,38_ = 40.956, *p* < 0.001 and *η*_*p*_^2^ = 0.789)], and post hoc analysis revealed that CSA was larger at 25% (CSA_25%_) and 75% (CSA_75%_) of its length compared with 50% (CSA_50%_) (*p* < 0.001) and larger at CSA_25%_ compared to CSA_75%_ (*p* = 0.035). Training increased mean PT CSA (F_1,18_ = 9.948, *p* = 0.001, *η*_*p*_^2^ = 0.443) and there was a time × group interaction (F_1,18_ = 6.037, *p* = 0.024, *η*_*p*_^2^ = 0.244, Table 3, Figure 5). In COL, PT CSA_25%_ and PT CSA_75%_ increased by 6.6 ± 7.3 mm^2^ (*p* = 0.03, *d* = 0.901 and *d* = 1.1) and 6.0 ± 5.4 mm^2^ (*p* = 0.01), respectively, with no change at PT CSA_50%_ (*p* = 0.15) after 12 weeks’ RT. There were no changes from pre‐ to post‐training at any location along the PT in PLA (F_1_,_20_ = 0.404, *p* = 0.539 and Figure 5).

### 3.5 Patellar Tendon Mechanical Properties

PT stress was higher in COL compared to PLA at baseline (*t*_18_ = 2.13, *p* = 0.047). There was a main effect of time (F_1,18_ = 35.04, *p* < 0.001) where PT stress increased post‐training, but no effect of group (F_1,18_ = 2.16, *p* = 0.159) and no group × time interaction (F_1,18_ = 2.97, *p* = 0.102). There was a main effect of time on PT strain, which decreased post‐training (F_1,18_ = 4.82, *p* = 0.041, *η*_*p*_^2^ = 0.443); however, there was no interaction (F_1,18_ = 3.38, *p* = 0.082).

**FIGURE 6:**
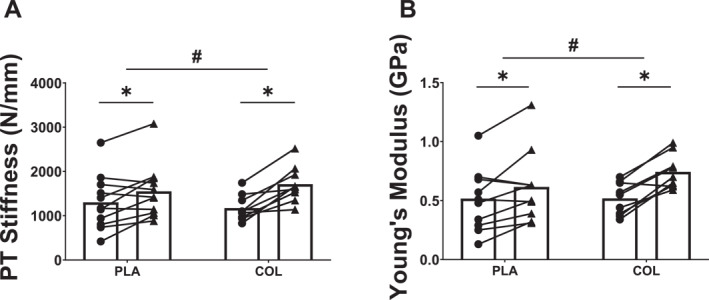
(A) Patellar tendon stiffness (N/mm) and (B) Young’s modulus (GPa) before (circles) and after (triangles) 12 weeks’ resistance training in COL (*n* = 9) and PLA (*n* = 11). *Greater than pre, # group × time interaction (*p* < 0.05).

Regarding patellar tendon stiffness (*k*), there were no differences between groups at baseline (*p* = 0.78). However, following the intervention, there was a main effect of time (F_1,18_ = 40.71, *p* < 0.001 and *η*_*p*_^2^ = 0.693) and no main effect of group (F_1,18_ = 0.113, *p* = 0.741), but there was a group × time interaction (F_1,18_ = 8.46, *p* = 0.009, *η*_*p*_^2^ = 0.320). Post hoc paired *t*‐tests revealed that *k* increased in both COL (*p* < 0.001 and *d* = 2.0) and PLA (*p* = 0.019 and *d* = 0.811), while an independent *t*‐test on the change from pre‐ to post‐training revealed that the increase in *k* was greater in COL (+661 ± 331 N/mm) compared with PLA (+247 ± 305 N/mm) (*t*_18_ = 2.91, *p* = 0.009, *d* = 1.31, Figure 6).

Similarly, there was a main effect of time on Young’s modulus (*ℰ*) (F_1,1__8_ = 30.73, *p* < 0.001, *η*_*p*_^2^ = 0.631) and no main effect of group (F_1,18_ = 0.398, *p* = 0.536), but there was a group × time interaction (F_1,18_ = 6.71, *p* = 0.018, *η*_*p*_^2^ = 0.271, Figure 6), where *ℰ* increased by 0.21 ± 0.13 GPa in COL (*p* < 0.001 and *d* = 1.83) and by 0.09 ± 0.13 GPa in PLA (*p* = 0.041 and *d* = 0.662). The pre‐to post‐training changes were greater in COL compared with PLA (*t*_18_ = 2.91, *p* = 0.048, *d* = 1.16).

## 4 Discussion

The objective of this study was to determine if supplementation with vitamin C‐enriched hydrolysed collagen augmented changes in PT morphological and mechanical properties in healthy, recreationally active, middle‐aged men. Consuming 30 g hydrolysed collagen enriched with 50 mg vitamin C twice per week after resistance training (RT) led to greater increases in PT CSA, stiffness and Young’s modulus compared to RT alone. This study is the first to demonstrate the effects of high‐intensity RT on tendon adaptations in middle‐aged men, and that these effects are enhanced by collagen supplementation.

The effects of RT on tendon stiffness are well established in young and older populations (Bohm et al. 2015; Lazarczuk et al. 2022). However, there is a gap in research concerning the effects of RT on tendon properties in *middle‐aged* individuals. To our knowledge, only one study has examined these effects in middle‐aged women, demonstrating no change in tendon stiffness with low‐load RT, specifically body weight squats (Kubo et al. 2003). Furthermore, the combined influence of nutrition and high‐intensity RT on tendon mechanical properties in ageing populations remains unexplored. Therefore, our study represents the first investigation into these effects in *middle‐aged* men.

In our study, participants in the placebo group (PLA) exhibited an increase in tendon stiffness without a corresponding increase in tendon size. This finding diverges from previous research in young populations, where RT‐induced tendon hypertrophy has consistently been observed (Quinlan et al. 2021; Seynnes et al. 2009; Kongsgaard et al. 2007; Massey et al. 2017). Notably, Quinlan et al. (2021) reported similar findings in older males, wherein patellar tendon mechanical properties improved without an increase in cross‐sectional area (CSA) following 8 weeks’ RT, mirroring the outcomes in our middle‐aged PLA group, who underwent RT without COL. Conversely, younger males in the same study experienced both tendon hypertrophy and increased stiffness (Quinlan et al. 2021). The increase in tendon stiffness observed in ageing individuals may be attributed to alterations in material properties, such as increased crosslinking and/or collagen fibril density (Heinemeier and Kjaer 2011), rather than tendon hypertrophy.

A meta‐regression analysis of RT studies that concurrently assessed tendon material properties and CSA suggested that an increase in modulus was the main contributor to short term changes in stiffness (Lazarczuk et al. 2022). However, the authors included various modes of chronic exercise with different contraction types and intensities, whereas *high‐intensity* RT (such as that employed in our study design) is known to be the key driver of tendon hypertrophy (Bohm et al. 2015). Conversely, we observed augmented tendon hypertrophy alongside enhanced stiffness in COL and, as reported previously, the hypertrophy occurred at the proximal and distal locations, where the tendon is closest to the osteotendinous junctions (Seynnes et al. 2009; Kongsgaard et al. 2007). This suggests that the tendon hypertrophy in COL was the primary factor contributing to the greater increase in stiffness.

RE stimulates collagen synthesis in tendon (Miller et al. 2005; Crossland et al. 2023), while collagen supplementation increases blood concentration of collagen amino acids (Lee, Tang, et al. 2023; Nulty et al. 2024; Alcock et al. 2019; Skov et al. 2019; Shaw et al. 2017). Crucially, Lee, Tang, et al. (2023) and Nulty et al. (2024) have shown that ingesting 30 g HC prior to RE increased whole body collagen synthesis in the hours following RE in both young (Lee, Tang, et al. 2023) and middle‐aged (Nulty et al. 2024) men. The greater bioavailability of key collagen amino acids may have stimulated greater whole body collagen synthesis by either supplying the crucial amino acids following a RE‐induced increase in collagen synthesis, or by stimulating collagen synthesis independently of RE via different signalling pathways (Mousavizadeh et al. 2020), or both. The PT hypertrophy in the COL group, compared to none in the PLA group, can be attributed to the different collagen synthesis responses in middle‐aged (Nulty et al. 2024) compared to young resistance‐trained men (Lee, Tang, et al. 2023). Firstly, middle‐aged men show no whole‐body collagen synthesis response to a single bout of RE (Nulty et al. 2024). However, 15 g HC ingestion seems to overcome this apparent anabolic resistance in middle‐aged men, with 30 g HC having an even greater effect. This contrasts with young men who show a clear augmentation in collagen synthesis following RE even without prior ingestion of HC (Lee, Tang, et al. 2023). This lack of collagen synthesis increase in middle‐aged men without HC ingestion may explain the absence of PT hypertrophy in our PLA group. In contrast, the COL group showed a 5.2% ± 4.9% increase in PT CSA, likely due to enhanced net collagen turnover from regular key amino acid ingestion in the COL group.

Two related studies suggest that collagen peptide supplementation combined with RT augments both patellar and Achilles tendon hypertrophy, with concomitant increases in patellar tendon stiffness (Jerger et al. 2023) but not Achilles tendon stiffness (Jerger et al. 2022). Our study’s distal PT hypertrophy in the COL group (+6.0% ± 5.2%) is less than the 10.7% increase reported by Jerger et al. (2023) in their collagen supplementation group but similar to the 6.5% increase in their RT‐only group. Total weekly training volume for the knee extensors in the study by Jerger et al. (2023), which involved younger participants, was similar to our study. Thus, age differences in study populations likely account for these discrepancies. Without collagen supplementation, older men may not experience the same hypertrophy from RT as younger men (Quinlan et al. 2021). Our data support the notion that tendon hypertrophy is blunted with ageing, starting in middle‐age, but 30 g hydrolysed collagen can restore the tendon’s ability to hypertrophy in response to RT. Finally, Balshaw et al. (2022) reported increased PT stiffness in young untrained men after 14 weeks’ RT, with no additional effects from 15 g·d^−1^ collagen ingestion, aligning with findings that 15 g collagen is insufficient to increase collagen synthesis after acute resistance exercise in young men, while 30 g does (Lee, Tang, et al. 2023).

In young female soccer players, both high‐intensity R and bodyweight training combined with 30 g collagen supplementation resulted in 15% and 18% increases in patellar tendon stiffness, respectively, in academy and professional athletes (Lee, Bridge, et al. 2023; Lee et al. 2024). By comparison, the observed 56% ± 29% increase in stiffness in our collagen group appears high. However, given that our participants were untrained prior to the study [unlike the athletes in both studies by Lee and colleagues (Lee, Bridge, et al. 2023; Lee et al. 2024)], and the lower tendon stiffness of the middle‐aged men at baseline, it is likely that they had greater capacity for adaptation compared to the younger athletes. Furthermore, our data align with the magnitude of change in tendon stiffness and Young’s modulus observed in previously untrained older men (Reeves et al. 2003; Quinlan et al. 2021). Additionally, it would be expected that *high‐intensity* RT would result in greater overload of the muscle‐tendon unit, leading to greater strain on the tendon and thus a stronger stimulus for adaptation (Arampatzis et al. 2007; Lavagnino et al. 2003).

Our finding suggested that RT alone increased muscle thickness is consistent with the body of research on adaptation to RT (Schoenfeld et al. 2017), yet there was no additional benefit of HC supplementation. In contrast, three recent studies have suggested muscle hypertrophy is enhanced by collagen supplementation (albeit in a very small number of measures) (Jerger et al. 2022; Balshaw et al. 2022). Although one study found that 15 g collagen protein can upregulate cellular pathways associated with muscle protein synthesis (Centner et al. 2022), the notion that collagen protein may augment muscular adaptations to RT is speculative. The amino acid profile of collagen is different to that of high quality proteins, such as whey, which has a relatively high composition of essential amino acids known to stimulate muscle protein synthesis (MPS) independently of RE (Tang et al. 2009). It is this difference in amino acid composition that probably explains why whey protein is a superior stimulant of MPS than collagen (Oikawa et al. 2020). This has also been demonstrated over time, whereby whey has produced superior outcomes to collagen after chronic RT (Jacinto et al. 2022). The habitual protein intake of participants in our study (1.1 g·kg^−1^ [before 30 g HC supplementation]) may have been insufficient to support optimal hypertrophy in this population compared with intakes ≥ 1.6 g·kg^−1^ (Nunes et al. 2022; Morton et al. 2018). Even though our HC supplement increased daily protein intake by ∼37% on training days in COL, this did not augment muscle hypertrophy, which further supports the notion that collagen is inferior to other protein sources to promote *muscle* adaptation to RT.

The increases in strength and power (e.g., isometric strength and RTD, 10‐RM back squat, CMJ and broad jump) observed in both groups can be explained by the effects of high‐intensity RT alone (Erskine et al. 2010). Supplementation with 30 g hydrolysed collagen did not augment adaptations in any of these tasks, which is consistent with recent reports in young men (Jerger et al. 2022; Balshaw et al. 2022), yet conflicts with studies in older men where collagen supplementation outperformed placebo in certain measures of strength (Zdzieblik et al. 2021, 2015). Interestingly, although COL supplementation led to larger improvements in tendon stiffness and Young’s modulus compared to RT alone, these changes were not accompanied by additional gains in explosive strength, power or RTD. This suggests that COL supplementation did not confer a further advantage over RT alone for improving RTD. A stiffer tendon should improve RTD and therefore translate to better dynamic exercise performance (Maffiuletti et al. 2016; Bojsen‐Møller et al. 2005). Although the intrinsic properties of tendon and muscle may contribute, the rate of force development in the knee extensors tends to be highly variable and is mainly driven by inter‐individual variability in neural factors (Folland et al. 2014). As our participants were previously untrained, variable increases in neuromuscular activation following our intervention may have outweighed any effect of muscle‐tendon unit stiffening. Our training intervention focused exclusively on high intensity RE, and thus participants were able to increase jump performance through increased force production primarily, without augmenting skill components.

### 4.1 Limitations

Our data were collected during a period of re‐opening following prolonged national lockdown due to the COVID‐19 pandemic. Therefore, we recruited participants from a previously untrained, yet active population, and it would not have been possible to recruit resistance trained men given the prolonged closure of gyms and leisure facilities. As a result, caution should be exercised when extrapolating these results to athletic populations. Moreover, although this is the first study to examine the combined effects of RT with collagen supplementation in middle‐aged adults, there is a reason to believe that middle‐aged women may not respond similarly to men (Magnusson et al. 2007), so further work is necessary to investigate the combined effects of collagen supplementation and RT on tendon properties in middle‐aged women. Finally, we acknowledge the limitations in our method of muscle size assessment (a single 38 mm wide sagittal view of muscle thickness) limits our ability to comment on regional muscle hypertrophy, although it should be emphasised that assessing the effects of RT and collagen supplementation on muscle size was not the primary purpose of this study.

### 4.2 Conclusion

This study demonstrates that 12 weeks’ high‐intensity resistance training with 30 g hydrolysed collagen supplementation augments patellar tendon CSA, stiffness and Young’s modulus more than resistance training alone. These findings have implications for exercise, nutrition and rehabilitation prescriptions in healthy middle‐aged men. Further research is required to elucidate if these effects can be replicated in *middle‐aged* women.

## Author Contributions

CDN and RME contributed to the conception and design of the study. CDN and KP were responsible for the data acquisition and supervision of the resistance training programme. All authors were responsible for data analysis. CN and RME were responsible for drafting the manuscript and all authors have approved the final version of the manuscript.

## Conflicts of Interest

The authors declare no conflicts of interest.

5

## Supporting information

Table S1

## Data Availability

Data described in the manuscript will be made available upon reasonable request.
