# Supplementary material for: Hydrolysed Collagen Supplementation Enhances Patellar Tendon Adaptations to 12 Weeks’ Resistance Training in Middle‐Aged Men
Source: Eur J Sport Sci. 2025 Mar 18;25(4):e12281. doi: 10.1002/ejsc.12281 (PMC11917387; doi:10.1002/ejsc.12281)
Supplement: Supplementary file 1 — Table S1 [file EJSC-25-e12281-s001.docx]

**Supplementary Table 1.** Composition of supplements used in COL (Hydrolysed Collagen) and PLA (Maltodextrin)

|  | **Hydrolysed Collagen** | **Maltodextrin** |
| --- | --- | --- |
|  | Per 100g | |
| Energy | 1530 kJ/360 kcal | 1700 kJ/400 kcal |
| Fat | 0g | 0 g |
| of which saturates | 0g | 0 g |
| Carbohydrates | 0g | 100 g |
| of which sugars | 0g | 0g |
| Protein | 90g | 0 g |
| Salt | 0.10g | <0.01 g |
